# Is healthcare really equal for all? Assessing the horizontal and vertical equity in healthcare utilisation among older Ghanaians

**DOI:** 10.1186/s12939-018-0791-3

**Published:** 2018-06-20

**Authors:** Vincent Dei, Miguel San Sebastian

**Affiliations:** 1 International SOS, Ghana, No. 2 Mankata Close Link, Airport Residential Area, Accra, Ghana; 2 Department of Public Health and Clinical Medicine, Epidemiology and Global Health Unit, Umeå University, SE-901 85 Umea, Sweden

**Keywords:** Healthcare utilisation, Horizontal equity, Vertical equity, Socioeconomic status, Older adults, Ghana

## Abstract

**Background:**

There is a lack of focused research on the older population in Ghana and about issues pertaining to their access to healthcare services. Furthermore, information is lacking regarding the fairness in the access to these services. This study aimed to ascertain whether horizontal and vertical equity requirements were being met in the healthcare utilisation among older adults aged 50 years and above.

**Methods:**

This study was based on a secondary cross-sectional data from the World Health Organization’s Study on global AGEing (SAGE) and adult health wave 1 conducted from 2007 to 2008 in Ghana. Data on 4304 older adults aged 50 years-plus were analysed. Bivariate and multivariable analyses were carried out to analyse the association between outpatient/inpatient utilisation and (1) socioeconomic status (SES), controlling for need variables (horizontal equity) and (2) need variables, controlling for SES (vertical equity). Odds ratios with 95% confidence intervals were calculated to analyse the association between relevant variables.

**Results:**

Horizontal and vertical inequities were found in the utilisation of outpatient services. Inpatient healthcare utilisation was both horizontally and vertically equitable. Women were found to be more likely to use outpatient services than men but had reduced odds of using inpatient services. Possessing a health insurance was also significantly associated with the use of both inpatient and outpatient services.

**Conclusion:**

Whilst equity exists in inpatient care utilisation, more needs to be done to achieve equity in the access to outpatient services. The study reaffirms the need to evaluate both the horizontal and vertical dimensions in the assessment of equity in healthcare access. It provides the basis for further research in bridging the healthcare access inequity gap among older adults in Ghana.

## Background

There has been an enormous interest in the issues of equity in access to healthcare nationally and internationally, with governments and international bodies working in concert to ensure universal coverage of health [1, 2]. Sub-Saharan economies such as Ghana [3] and South Africa [4] have taken steps towards ensuring equitable access to healthcare in the form of health insurance schemes.

A major aim of ensuring equity in healthcare provision policies of many governments, is to provide equitable access (or utilisation) so that all individuals have equal access to at least, basic healthcare services based solely on their health needs. Healthcare access has also been viewed by some as how individuals are empowered to use healthcare where factors such as availability, affordability and acceptability directly affect access to care [5]. Healthcare utilisation, as a proxy of healthcare access conceptualised by Andersen, is determined by three broad sets of factors, namely predisposing individual socio-cultural and demographic factors, enabling factors and need factors [6, 7]. This is the adopted approach in this study.

Horizontal and vertical equity concepts are however key considerations in the better understanding of how healthcare is utilised [8, 9]. The ability to identify specific disadvantaged social groups based on these two principles is central to the policy-making processes across many health systems. This is crucial to removing any systematic disparities that portend for poor health or providing adequate measures to cushion vulnerable populations such as older persons against adverse health outcomes.

### Ageing and healthcare utilisation in Ghana

There is a growing population of older persons in Low-and-Middle Income countries [10] with a projected increase in the number of adults 60 years plus (elderly) in Sub-Saharan Africa from 4.8% of total population in 2015 to 7.5% by 2050 [11]. This ageing raises concerns mainly because factors such as illiteracy, living in rural areas with poor infrastructure, lack of employment as well as erosion of family structures put the elderly in a very vulnerable position to access health in the West African sub-region [12]. This reality among other factors, has sparked the need for health sector reforms in the sub-region towards achieving a universal health coverage [13].

With increasing socioeconomic development, Ghana’s population has been experiencing a trend of decreasing infectious diseases with increasing longevity but with an increment in the proportion of chronic and age-related diseases [14]. The need for healthcare usage is likely to increase. How this care is utilised however could be influenced by factors such as educational level [15, 16], socioeconomic status [17, 18] the presence of chronic illness [16, 17, 19], family support [20] and access to health insurance [21], the distribution of which can occasion inequity.

The implication is that Ghana’s health system must adapt to incorporate policies in healthcare delivery that put the elderly in the limelight whilst still aiming to reduce mortality at younger ages. Policies on health financing mechanisms for instance must be cognisant of the changing age structure if access to care among the older population is to be improved. Other policies such as the Government of Ghana’s National Ageing Policy [22] which aims at improving the general well-being of older persons, would, however, need constant empirical feed-in to make any solutions relevant.

Ghana’s healthcare system is made of public, private (both for-profit and not-for-profit) and other services such as traditional medical practitioners. Access to healthcare is however predominantly through public facilities. The health sector as a whole is currently funded mainly through public funds, household contributions (including out-of-pocket spending), inter-governmental transfers and external supports in grants and loans [23].

Post-independence, access to the public health system required no payment at the point of service, being funded by general taxes and donor support. In 1985 however, the introduction of user-fees represented a major barrier to accessing care and subsequently reduced the use of health services by the very poor and the elderly [24]. In order to improve access to healthcare and achieve universal coverage for all Ghanaians, the National Health Insurance Scheme (NHIS) was instituted in 2003 [25] and funded through taxes, premiums and donor support. The scheme is however currently cash-strapped and public discourse regarding increasing taxes to generate more revenue appears negative so far [26].

Despite its challenges, Ghana was the first in Sub-Saharan Africa to have embarked on an ambitious plan towards providing universal health coverage for its citizens through National Health Insurance [27]. Other Sub-Saharan Africa nations such as South Africa and Tanzania have taken similar steps but with much less coverage goals [28]. In West Africa, Ghana’s edge over other countries in the sub-region such as its neighbour Nigeria, in healthcare access and major health indices (infant mortality, under-5 mortality and life expectancy) has been documented [29]. Ghana is therefore a good example in examining whether the strides made towards universal coverage translate into equity in healthcare use among older persons in terms of outpatient and inpatient visits.

Access to healthcare services in the general Ghanaian population have been found to be pro-rich, even in public health facilities supported by public funds [30, 31]. As part of efforts to improve access to healthcare among the aged, persons over 70 years are exempt from paying any premiums to the NHIS. In addition, older poor persons 65 + years are entitled to free enrolment in the NHIS under the Ghana government’s social protection programme, Livelihood Empowerment Against Poverty (LEAP) [32]. The Health Insurance Authority emphasises its strive to achieve both horizontal and vertical equity regarding healthcare access in its operations [25]. However, the question of whether this is being achieved and to what extent among vulnerable groups like the older population, unfortunately, remains largely unanswered.

Factors such as gender, rural-urban and poor-rich gaps have featured prominently in Ghana’s healthcare policies and research has focused on the use of health services with particular attention to children and pregnant mothers. There is, however, lack of focused research concerning the older population in Ghana, generally, and specifically about their access to healthcare services. Of the few studies based on a large dataset that have examined healthcare services utilisation among older adults, the equity dimensions have been less explored [33]. In studies that attempted to deal with utilisation equity, the assessment has usually been limited to horizontal equity [21, 34]. To answer the question whether healthcare is really equal for all, however, demands equal attention to both equity dimensions [9, 35].

In this paper, we assessed whether both the horizontal and vertical equity requirements regarding healthcare utilisation were being met in the elderly population of Ghana. The study helps to identify some critical issues of equity in healthcare use among this population sub-group and opportunities for further studies in this area. We also highlight some of the possible issues health policymakers may have to contend with regarding where resources might be better focused.

## Methods

### Study design

This was a cross-sectional survey based on a secondary data from the wave 1 of WHO multi-country longitudinal Study on Global Ageing (SAGE) carried out from 2007 to 2008 in 10 administrative regions in Ghana [36, 37]. A nationally representative sample was taken from individuals aged 50 years and above in a stratified multi-stage cluster design. A detailed explanation of this procedure is available elsewhere [37].

### Study population

All of Ghana’s ten administrative areas and whether an area was urban or rural were used as the units of stratification in the multi-stage cluster design. Individual-level and household data were collected where each household completed a questionnaire and individuals aged 50 years plus in a household were interviewed. Persons found unable to respond to the individual questionnaires had them administered through a proxy respondent [37].

Data were collected from 5573 individuals and 5269 households by a team of trained interviewers using standardised questionnaires that were translated into the local languages Akan, Ga or Twi in a face-to-face contact [37]. This study was however limited to a total of 4304 households and individual data on people aged 50 years and above who were the sub-population of interest to this study. Further information on WHO SAGE Wave 1 study can be found elsewhere [36, 37].

### Measures

The predisposing factors considered were age, sex and residence (sociodemographic); the enabling factors wealth quintile, education and health insurance; and the health need factors of self-rated health status and morbidity level. Outpatient and inpatient services use were the outcomes in this study.

#### Independent and control variables

##### Predisposing factors

Age of respondents was described by the categories “50–59”, “60–69”, “70–79”, “80+” year age groups. Sex was coded as “male” or “female”. The original WHO questionnaires made no distinctions in terms of gender roles. All areas that had been legally proclaimed as urban were designated “urban” and all others without any such legal status were considered “rural” in assessing the variable “residence”.

##### Enabling factors

Education is an important determinant of healthcare utilisation [15] and a sensitive indicator of socioeconomic status (SES) among older persons [38]. Respondents’ education level was categorised as none, primary, secondary and tertiary.

As indicated elsewhere by the WHO SAGE principal investigators [36, 37], a two-step random effect probit model was used to generate wealth quintiles based on respondents’ living characteristics, access to basic amenities and ownership of durable goods for 21 assets. Quintile five and quintile one represented the wealthiest and poorest fifths of respondents respectively.

The health insurance status of respondents was also assessed and coded as 1 if the participants referred to having the insurance.

##### Health need factors

In this study, health needs were assessed using two variables, namely, self-rated health status and self-reported morbidity levels.

Although self-health rating is a subjective way of assessing health and may be unstable, several scientific works have indicated that it is a good predictor of healthcare utilisation [39] and health needs [40] with good test-retest reliability [41]. Self-rated health was assessed with the question “In general how would you rate your health today?”. Responses were coded on a five-point Likert-type scale from “1 – very good” to “5- very bad”.

In assessing the level of morbidity, a composite measure of five chronic diseases was derived by summing the number of these diseases that respondents reported. Diseases considered were hypertension, diabetes, arthritis, asthma and depression based on their context relevance. Respondents were categorised into those with none for no disease, single for only one ailment and comorbidity for two or more diseases.

#### Dependent variables

Two outcome variables were used to capture healthcare use. To assess the outpatient use of healthcare services, respondents were asked whether, over the preceding 12 months, they had received any healthcare, not including an overnight stay in a hospital or long-term care facility. The assessment of inpatient services use was based on whether participants stayed overnight in a hospital or long-term care facility in the preceding 3 years. A longer recall period for inpatient services usage was needed to generate enough data for any meaningful analysis.

#### Other variables

To ascertain the most widely used healthcare service, the study participants were asked the question “Thinking about health care you needed in the last 3 years, where did you go most often when you felt sick or needed to consult someone about your health?” The responses were categorised into “private”, “public”, “charity”, “others”, “over 3 years”(those who had not used any services within the past 3 years). “Others” referred to other services such as pharmacies and traditional medical practitioners.

### Data analysis

To ensure nationally representative data, individual and household level weights determined by the selection probabilities at each sampling stage were applied to the dataset. Post-stratification correction weights by region and locality based on the 2010 housing and population census projections of the Ghana Statistical Service were also applied [36, 37]. This was to correct for any sampling biases that would have been introduced as a result of using the Census Enumerated Areas of the 2000 Population and Housing Census with updated household listings in 2007 as the sampling frame. Further explanation of this is provided elsewhere by Biritwum et al. [37]. Descriptive statistics were used to summarise the population characteristics using counts and weighted percentages. Pearson’s chi-square was used to analyse sex differences for the various categorical variables.

Bivariate and multivariable logistic regression analyses were conducted to examine any crude and adjusted relationships respectively between each of the independent and dependent variables. To identify multicollinearity among the variables, variance inflation factors were calculated. For each of inpatient and outpatient utilisation outcomes, three models were developed. Model 1 examined the relationship between the independent variables and dependent variables with wealth quintile as the main SES predictor variable and Model 2 with educational level as the main SES predictor variable. Model 3 is the full model that examined the combined effect of both SES predictor variables on both outpatient and inpatient healthcare access, whilst controlling for the other variables. Six models in all were thus developed. The inclusion of the variables in each module was based on previous research and relevance to the study objectives. The calculated variance inflation factors (VIF) for all the variables included in the models were between 1 and 2 indicating acceptable levels of multicollinearity.

To assess horizontal equity, the association between SES variables and healthcare use was examined whilst controlling for health needs (self-rated health, morbidity), sociodemographic and predisposing factors (age, sex, residence) and health insurance in the full model. In assessing vertical equity, the associations between health need variables and healthcare access were examined whilst adjusting for SES and other variables. Odds ratios with 95% confidence interval were computed to determine the strength of the associations between variables. Goodness-of-fit for the models was tested using the Hosmer-Lemeshow test for complex survey data [42] where a *p*-value above alpha = 0.05 indicated a model appropriately fitting the data. All statistical analyses were carried out using STATA 13.1 statistical software.

### Ethical considerations

Approval for the primary study was granted by the Ethical Review Board of the World Health Organization with additional approval obtained from the University of Ghana Medical School’s Ethical and Protocol Review Committee [34]. Informed consent was also sought and confidentiality ensured. Access to the secondary data from WHO Multi-Country Studies Data Archive was granted by WHO after all the necessary pre-conditions were met.

## Results

### Population characteristics

Of 4304 records of subjects aged 50 years and above who undertook the study, analyses were restricted to observations with complete data for the relevant variables. Table 1 describes the general characteristics of the study population with applied study population weights, stratified by sex. In all, 52.47% (*n* = 2249) of the respondents were males and 47.53% (*n* = 2055) females. Most of the respondents (39.76%) were aged between 50 and 59 years with the least represented group being those 80 years and over (9.7%). There was no significant difference between males and females in terms of age groups (*p* = 0.45). Participants were also mostly from rural communities (58.9%).

**Table 1:** Weighted percentages of demographic, socioeconomic and health characteristics

Characteristic (N)	Male %	Female %	Total % (n)	*p*-value
Age in years (4304)				
50–59	40.57	38.86	39.76 (1711)	0.45
60–69	26.94	28.06	27.47 (1182)	
70–79	22.25	23.97	23.07 (993)	
80+	10.24	9.11	9.70 (418)	
Residence (4304)				
Rural	59.31	58.44	58.9 (2535)	0.67
Urban	40.69	41.56	41.1 (1769)	
Education level (4278)				
None	43.38	65.48	53.91 (2306)	0.00
Primary	22.50	20.10	21.36 (914)	
Secondary	29.04	12.42	21.12 (904)	
Tertiary	5.09	1.99	3.61 (154)	
Wealth quintile (4299)				
1 (Lowest)	16.32	20.31	18.22 (783)	0.00
2	17.22	21.16	19.09 (821)	
3	19.61	21.41	20.46 (880)	
4	21.52	19.72	20.66 (888)	
5 (Highest)	25.34	17.4	21.56 (927)	
Health insurance (4303)				
No	61.08	62.63	61.82 (2660)	0.46
Yes	38.92	37.37	38.18 (1643)	
Outpatient use (4142)				
No	41.41	34.4	38.02 (1575)	0.00
Yes	58.59	65.6	61.98 (2567)	
Inpatient use (4103)				
No	87.81	89.99	88.86 (3646)	0.07
Yes	12.19	10.01	11.14 (457)	
Healthcare facility type (3954)				
Public	49.54	55.51	52.41 (2072)	0.02
Private	14.86	12.61	13.78 (545)	
Charity	4.46	5.03	4.73 (187)	
^a^Others	25.38	22.16	23.83 (942)	
Over 3 yrs	5.77	4.69	5.25 (208)	
Health status (4300)				
Very good	4.86	3.09	4.02 (173)	0.00
Good	41.52	32.46	37.21 (1600)	
Moderate	38.9	44.68	41.65 (1791)	
Bad	12.78	17.16	14.86 (639)	
Very Bad	1.94	2.61	2.261 (97)	
Morbidity (4304)				
None	63.05	52.07	57.83 (2489)	0.00
Single	27.88	31.16	29.41 (1266)	
Comorbidity	9.12	16.78	12.76 (549)	

^a^Others -refers to other health services like pharmacy and traditional medicine; (N) – total sample size analysed

Over half of the study subjects (53.91%) had no formal education of which females were in the majority (65.48% of females). Less than 4% have had a tertiary education with more than twice as many males as females achieving this. This sex difference in attainment of the highest education level appeared statistically significant (*p* < 0.01).

**Fig. 1:**
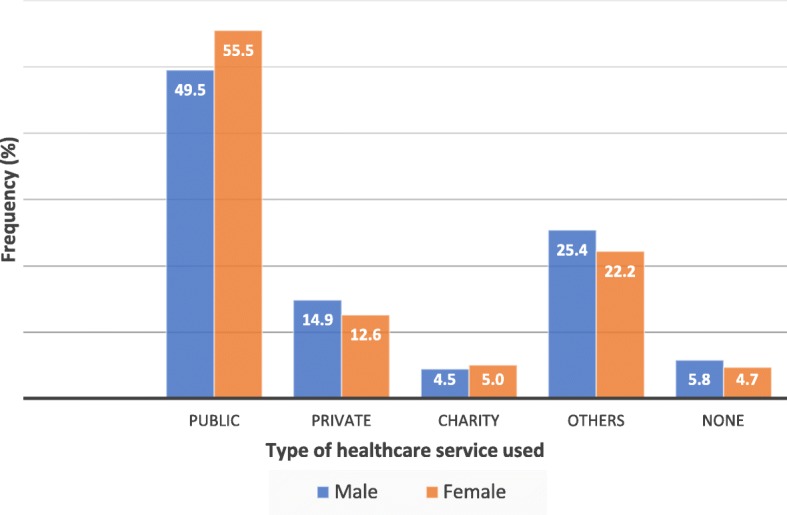
Distribution of healthcare utilisation according to sex in the previous 3 years. “Others” referred to other services such as pharmacies and traditional medical practitioners

Most of the respondents (61.82%) were not insured (Table 1). Regarding outpatient services use, about two-thirds (61.98%) indicated using the services over the previous 12-month period. On the contrary, fewer (11.14%) had used inpatient services over the preceding 3-year period. There appeared to be a significant sex difference in the use of outpatient services but not of the inpatient services (*p* < 0.01 and *p* = 0.07 respectively). Public health services were the most frequently used (52.41%) with about 5% of the population not needing healthcare services over the preceding 3-year period. Figure 1 further illustrates the general distribution of respondents’ outpatient and inpatient healthcare services utilisation.

Overall, the majority thought they were in a moderate to very good health state (82.88%). Relatively more males rated themselves to be in a better health than females and this difference appeared statistically significant (*p* < 0.01). In terms of self-reported prevalence of chronic diseases, over half (57.83%) reported no chronic diseases, a third (29.41%) a single disease and a tenth (12.76%) had comorbidities.

### Crude logistic regression estimates

Tables 2 and 3 show the results of bivariate analyses indicating the crude estimates of the strength of association between variables and both outpatient and inpatient utilisation.

**Table 2:** Logistic regression models of the association between outpatient services utilisation and SES or health need

Variable	Crude model	Adjusted models		
	OR [CI]	Model 1^a^	Model 2^b^	Model 3^c^
		OR [95% CI]	OR [95% CI]	OR [95% CI]
Age in years				
50–59	1	1	1	1
60–69	1.26* [1.05,1.50]	1.18 [0.98,1.41]	1.25* [1.04,1.51]	1.26* [1.05,1.52]
70–79	1.48*** [1.21,1.82]	1.22 [0.97,1.52]	1.41** [1.11,1.78]	1.40** [1.10,1.77]
80+	1.71*** [1.27,2.30]	1.38 [0.98,1.94]	1.73** [1.23,2.42]	1.69** [1.20,2.37]
Sex				
Male	1	1	1	1
Female	1.35*** [1.16,1.57]	1.38*** [1.18,1.62]	1.49*** [1.26,1.76]	1.53*** [1.29,1.80]
Residence				
Rural	1	1	1	1
Urban	1.43** [1.14,1.79]	1.08 [0.84,1.39]	1.21 [0.95,1.54]	1.05 [0.81,1.35]
Health insurance				
No	1	1	1	1
Yes	2.22*** [1.85,2.68]	1.90*** [1.57,2.31]	1.99*** [1.64,2.40]	1.87*** [1.54,2.26]
Education level				
None	1	–	1	1
Primary	1.28* [1.03,1.59]	–	1.55*** [1.22,1.97]	1.42** [1.11,1.81]
Secondary	1.50***[1.19,1.90]	–	1.92*** [1.46,2.53]	1.68*** [1.27,2.23]
Tertiary	1.95**[1.29,2.97]	–	1.92** [1.24,2.98]	1.60* [1.00,2.55]
Wealth quintile				
1 (Lowest)	1	1	–	1
2	1.47** [1.13,1.90]	1.46** [1.12,1.91]	–	1.41* [1.07,1.85]
3	2.02*** [1.59,2.57]	1.99*** [1.56,2.53]	–	1.84*** [1.44,2.35]
4	2.12*** [1.63,2.75]	2.01*** [1.54,2.62]	–	1.80*** [1.37,2.37]
5 (Highest)	2.64*** [1.98,3.51]	2.48*** [1.83,3.36]	–	2.09*** [1.52,2.88]
Health status				
Very good	1	1	1	1
Good	0.42*** [0.27,0.67]	0.39*** [0.24,0.61]	0.39*** [0.25,0.62]	0.39*** [0.24,0.61]
Moderate	0.67 [0.42,1.06]	0.58* [0.36,0.93]	0.57* [0.35,0.92]	0.58* [0.36,0.94]
Bad	0.95 [0.58,1.57]	0.83 [0.50,1.39]	0.80 [0.48,1.34]	0.84 [0.50,1.40]
Very bad	1.80 [0.81,3.97]	1.63 [0.70,3.81]	1.56 [0.65,3.74]	1.63 [0.69,3.86]
Morbidity				
None	1	1	1	1
Single	1.45*** [1.20,1.75]	1.21* [1.00,1.46]	1.26* [1.04,1.51]	1.20 [1.00,1.45]
Comorbidity	1.72*** [1.30,2.26]	1.21 [0.91,1.62]	1.24 [0.91,1.61]	1.16 [0.87,1.55]
F-statistic	–	1.02	0.44	1.52
^d^Goodness-of-fit (Prob > F)	–	0.4264	0.9125	0.1436

OR – Odds Ratio with reference group as 1; CI – 95% Confidence Interval; *p < 0.05, **p < 0.01, ***p < 0.001, p is *p*-value

^a^Model 1 - wealth quintile is the main SES variable, adjusted for sociodemographic factors, insurance and health needs (health status and morbidity)

^b^Model 2 - education is the main SES variable, adjusted for sociodemographic factors, insurance and health needs (health status and morbidity)

^c^Model 3 - the full model, controlling for all variables. (SES, sociodemographic factors, insurance and health needs)

^d^Goodness-of-fit – Hosmer-Lemeshow test with greater values indicating better models

**Table 3:** Logistic regression models of the association between inpatient services utilisation and SES or health need

Variable	Crude model	Adjusted models		
	OR [CI]	Model 1^a^	Model 2^b^	Model 3^c^
		OR [95% CI]	OR [95% CI]	OR [95% CI]
Age in years				
50–59	1	1	1	1
60–69	1.06 [0.81,1.39]	0.96 [0.73,1.27]	0.93 [0.70,1.24]	0.93 [0.71,1.24]
70–79	1.14 [0.83,1.56]	0.93 [0.68,1.28]	0.90 [0.64,1.28]	0.90 [0.63,1.27]
80+	1.43 [0.91,2.27]	1.08 [0.68,1.72]	1.02 [0.64,1.61]	1.01 [0.64,1.60]
Sex				
Male	1	1	1	1
Female	0.80 [0.63,1.02]	0.75* [0.59,0.96]	0.74* [0.56,0.93]	0.73* [0.57,0.95]
Residence				
Rural	1	1	1	1
Urban	1.58** [1.20,2.08]	1.44* [1.07,1.95]	1.60** [1.19,2.14]	1.47* [1.08,1.98]
Health insurance				
No	1	1	1	1
Yes	1.42** [1.11,1.83]	1.28* [1.01,1.66]	1.36** [1.07,1.74]	1.31* [1.02,1.69]
Education level				
None	1	–	1	1
Primary	0.93 [0.67,1.28]	–	0.92 [0.65,1.29]	0.89 [0.64,1.24]
Secondary	1.03 [0.75,1.42]	–	0.91 [0.64,1.29]	0.85 [0.59,1.21]
Tertiary	0.77 [0.41,1.47]	–	0.60 [0.31,1.18]	0.53 [0.27,1.04]
Wealth quintile				
1 (Lowest)	1	1	–	1
2	1.24 [0.82,1.88]	1.24 [0.81,1.90]	–	1.26 [0.83,1.93]
3	1.15 [0.77,1.73]	1.09 [0.74,1.61]	–	1.13 [0.76,1.67]
4	1.38 [0.91,2.07]	1.20 [0.78,1.83]	–	1.27 [0.83,1.94]
5 (Highest)	1.80** [1.20,2.70]	1.43 [0.91,2.23]	–	1.56 [0.99,2.45]
Health status				
Very good	1	1	1	1
Good	1.82 [0.96,3.48]	1.90 [0.99,3.66]	1.88 [0.99,3.57]	1.88 [0.98,3.61]
Moderate	1.84 [0.95,3.57]	1.94 [0.98,3.86]	1.83 [0.96,3.60]	1.86 [0.94,3.70]
Bad	3.75*** [1.82,7.75]	4.13*** [1.94,8.79]	3.88*** [1.84,8.17]	4.01*** [1.89,8.85]
Very bad	3.32* [1.31,8.38]	3.67** [1.42,9.51]	3.43* [1.35,8.74]	3.60** [1.40,9.25]
Morbidity				
None	1	1	1	1
Single	1.13 [0.86,1.49]	0.99 [0.73,1.34]	1.04 [0.77,1.39]	1.02 [0.75,1.38]
Comorbidity	1.63*** [1.23,2.16]	1.39* [1.02,1.90]	1.42* [1.05,1.92]	1.39* [1.02,1.89]
F-statistic	–	1.33	0.59	0.53
^d^Goodness-of-fit (Prob > F)	–	0.2247	0.8044	0.8506

OR – Odds Ratio with reference group as 1; CI – 95% Confidence Interval; *p < 0.05, **p < 0.01, ***p < 0.001, p is *p*-value

^a^Model 1 - wealth quintile is the main SES variable, adjusted for sociodemographic factors, insurance and health needs (health status and morbidity)

^b^Model 2 - education is the main SES variable, adjusted for sociodemographic factors, insurance and health needs (health status and morbidity)

^c^Model 3 - the full model of controlling for all variables (SES, sociodemographic factors, insurance and health needs)

^d^Goodness-of-fit – Hosmer-Lemeshow test with greater values indicating better models

For outpatient utilisation, we observed a positive and statistically significant association between age, sex, residence, educational achievement, wealth quintile, having health insurance and morbidity level (Table 2). Respondents who rated their health as “good” were also less likely to use outpatient services when compared with those with “very good” health (OR = 0.42; 95% CI: 0.27,0.67).

For inpatient utilisation, Table 3 shows that age, sex and educational level did not seem to have a significant association. Living in an urban area, belonging to the highest wealth quintile, possessing a health insurance, having a “bad” or “very bad” health status and reporting two or more diseases were however significantly associated with the use of inpatient healthcare.

### Multivariable logistic regression estimates

#### Horizontal equity

##### Outpatient services

From Table 2 (Model 2) we observed a significant and positive trend with those attaining tertiary level education being about 92% more likely to use outpatient services compared to those without education (OR = 1.92; 95% CI: 1.24,2.98). This positive effect of education on care usage remained significant even after adjusting for the impact of wealth (Model 3). Those with tertiary and secondary education were 60% (OR = 1.60; 95% CI: 1.00,2.55) and 68% (OR = 1.68; 95% CI: 1.27,2.23) respectively more likely to access outpatient care than those with no education when health needs were considered.

Table 2 also shows that the statistically significant and general positive trend where the wealthiest accessed more outpatient care as seen in the crude model was sustained even after controlling for health needs and other factors (Models 1 and 3). Those in the highest wealth quintile had 2.09 times (95% CI: 1.52,2.88) the odds of those in the lowest quintile to utilise outpatient services in the fully adjusted model (Model 3), a pro-rich inequity.

The use of outpatient services was thus observed to be significantly associated with both education and wealth after adjusting for health needs, indicating horizontal inequity.

##### Inpatient services

We observed a negative gradient in utilisation comparing the different levels of educational achievement to those having no education (Table 3). Those with tertiary education tended to be 47% less likely to access inpatient services (OR = 0.53; 95% CI: 0.27,1.04) after controlling for all variables (Model 3). This was however not statistically significant.

The statistically significant association between being in the highest wealth quintile and access to inpatient care that was observed in the crude model was not sustained after controlling for health needs and other factors (OR = 1.56; 95% CI: 0.99,2.45) (Model 3).

Overall, we found education and wealth not significantly associated with inpatient healthcare access after adjusting for health needs indicating that horizontal equity was present.

#### Vertical equity

##### Outpatient services

Relative to those reporting “very good” health, respondents who reported “good” and “moderate” health were less likely to access outpatient care (Table 2, Model 3). This was statistically significant. Those in “bad” health state also had insignificantly reduced odds of using services (Table 2, Models 1–3). Although those reporting “very bad” health state were about 63% more likely to use services than those in “very good” health (OR = 1.63; 95% CI: 0.69,3.86) but this was not statistically significant.

A statistical significance of the initially observed gradient of higher odds for care utilisation for a higher number of morbidities in the crude model was lost after adjusting for education and wealth (Table 2, Model 3). Overall, no statistically significant association between self-rated health and morbidity level and outpatient healthcare services use was observed, reflecting vertical inequity.

##### Inpatient services

After controlling for wealth and other factors (Model 1), respondents reporting “bad” and “very bad” health states were significantly more likely to access healthcare relative to those in “very good” health. Upon further adjustment for education, those in “bad” health (OR = 4.01; 95% CI: 1.89,8.85) and “very bad” health (OR = 3.60; 95% CI: 1.40,9.25) were still more likely to use inpatient healthcare services (Model 3) indicating vertical equity.

After adjusting for SES and other factors, respondents were observed to be slightly more likely for admission if they had one or more chronic diseases, and this was statistically significant for having a comorbid state (Models 2 and 3). Overall, vertical equity was observed in the use of healthcare for inpatient services among the study population.

#### The relationship of other variables with the use of healthcare services

For the predisposing factor age, a significant positive gradient in the odds of using outpatient care was observed (Table 2, Models 2 and 3). No statistically significant association was however found between age and the use of inpatient care (Table 3, Models 1–3).

Females had significantly higher odds than males in the use of outpatient care after controlling for sociodemographic factors, insurance, health status and wealth (OR = 1.38; 95% CI: 1.18,1.62) (Table 2, Model 1). The strength of this association slightly increased after additional adjustment for educational achievement (OR = 1.53; 95% CI: 1.29,1.80) (Model 3). On the contrary, females were 27% less likely to use inpatient services than males after controlling for all other variables (OR = 0.73; 95% CI: 0.57,0.95) (Table 3, Model 3).

Urban dwellers also had more access to inpatient services as against rural dwellers (Table 3, Models 1–3) although not in the case of outpatient care access (Table 2). Possessing a health insurance was also found to be an important enabling factor as it was positively associated with both inpatient and outpatient services usage (Tables 2 and 3, Models 1–3).

## Discussion

In this study, we sought to ascertain whether healthcare was equal for all older Ghanaians by examining how well horizontal and vertical equity concepts were operationalised in terms of access to healthcare. The results clearly pointed to the fact that, generally, whilst inpatient care was largely need-driven, the use of outpatient services was pro-rich in nature.

Our findings show that wealth and education are strong enabling factors in outpatient care usage and these drive the observed horizontal inequity in the use of these services. This is consistent with other studies that have found SES as a major determinant of outpatient services usage. For example, Peltzer et al. observed in their multi-country study involving six LMICs that the poorest quintile of older persons were about 30% less likely to use outpatient services [21]. The observed horizontal inequity supports the view held by researchers such as Phelan et al. that individuals use resources (wealth or knowledge) as strategies to avoid or minimise health risks [43]. It is possible that older Ghanaians with the highest SES are more enlightened about their health, are better equipped financially and can more readily access outpatient care services. As asserted by Saeed et al., given the Ghanaian cultural context where the rich usually have lifestyle problems related to sedentary lifestyle, lack of exercise and fatty meals, they may be more prone to chronic diseases that would necessitate higher outpatient care use [34]. That notwithstanding, considering the observed independent impact of education on outpatient care usage, it might be useful, even in situations where financial barriers to care are removed, to implement policies that make older people or their caregivers aware of the importance of using these services when needed.

In our study, no association between self-rated health or morbidity level and the use of outpatient services was found, which is contrary to the observation of other researchers [21]. This finding suggests that outpatient care access is predominantly influenced by other factors such as socioeconomic and not need. This is not surprising for a system that is not truly universal in healthcare coverage.

Studies suggest that SES has a positive impact on the use of inpatient services in older populations [18, 34]. In this current study, however, we found no statistically significant association between inpatient care use and either education or wealth. Consistent with our finding, Wong and Diaz also observed horizontal equity in the hospitalisation of older Mexican adults [19].

The observed horizontal equity in inpatient care access could partially be attributed to the fact that the rich are able to access early outpatient care and prevent complications, judging from the significant horizontal inequity in the access to outpatient services. They may, therefore, be in a relatively lesser need for inpatient care compared to outpatient care. It is also possible that the rich may be adopting more preventive and health promotive initiatives. The poor and less educated would, however, have fewer resources both in terms of finances and information and thus may delay in making decisions to access care until it reaches a critical stage.

After controlling for SES, we also found self-rated health and morbidity level to be significantly associated with the use of inpatient services in sharp contrast to what was observed in outpatient care usage.. This could partly be due to the fact that, the decision to admit to bed is one that is usually taken by a healthcare giver who would objectively assess the clinical state and hence the level of need to decide whether an overnight stay at a health facility is warranted. These findings buttress the observations of other researchers that found vertical equity in hospitalisation in terms of self-rated health [34, 44] and morbidity level [15, 21]. On the contrary, Roy and Chaudhuri indicated in their study of older Indians that subjective health status had no major impact on hospitalisations in that population [18].

Ghana’s National Policy on Ageing [22] represents an important step towards ensuring better health in general for older Ghanaians. Unfortunately, not much has been achieved yet in terms of its implementation. Currently, no clear standards and guidelines on older persons care provision and rehabilitation services exists nor is there a clear implementation plan on how health staff would integrate geriatric care into healthcare delivery at any level [45]. The guidelines can, for example, provide a framework for prioritising the needs of older persons. These should hopefully provide some framework for achieving vertical equity in outpatient utilisation.

Consistent with many studies in LMIC on inpatient and outpatient use [15, 18, 19, 21, 34], we observed that females used significantly more outpatient services than males but were less likely to be admitted for an inpatient care. Factors such as gendered differences in illness construction, care-seeking behaviour and social norms have been alluded to as possible reasons [21]. It might be the case that older Ghanaian women are more proactive towards early care-seeking such that they have comparatively fewer complications that would later warrant hospitalisation.

Increasing age was also found to be a predictor of outpatient use but not for inpatient care. Albanese et al., however, did not find any association between age and the use of community services in a large survey of nine LMICs [15]. As it is inevitable that LMICs like Ghana will continue to expand in the population of older adults, it might be worthwhile encouraging health promotion and preventive health activities among the older population. This may not only serve to reduce the cost of curative healthcare but could potentially help reduce healthcare disparities [46].

Health insurance has been found to be a strong enabling factor in accessing care, especially in settings where no universal coverage schemes exist and out-of-pocket payments seem to be relatively high [15]. We found that possessing health insurance was significantly associated with both increased inpatient and outpatient care access. It is, therefore, laudable that Ghana’s national health insurance scheme (NHIS) currently covers all citizens 70 years and above and formal sector workers 60 years and above. It is, however, worth considering the critical mass of those between 60 and 69 years in the informal sector. Most people in this group of older Ghanaians probably have no pension schemes or regular income and thus constitute a disadvantaged group that must be reached. Because they constitute the second most populous group of older adults 50 years and above (Table 1) it is possible that any intervention that tries to remove financial barriers to accessing care may yield positive results in promoting equity among the older population. In this regard, it is important to thoroughly debate and resolve the issues surrounding the NHIS funding for there to be any hope of its expansion to include the group of older persons currently not catered for.

We also observed that the area of residence was only important in the access to inpatient services where urban dwellers were at an advantage over those living in rural areas. In the Ghanaian context, chronic or lifestyle diseases have been noted to be more prevalent among those with relatively higher SES [47, 48] who mostly reside in urban areas. It is possible that these individuals may have more health risks warranting admissions than those in rural areas. Healthcare services are also usually concentrated in the urban areas where they are relatively better equipped in terms of manpower and logistics [45] and therefore serve as referral facilities and are able to offer more inpatient services. The observed disparity in access to inpatient care may thus also be because of disparities in resource allocation.

### Strengths and limitations

The main strength of this study draws from the fact that, it is among the very few that has looked at the use of healthcare among older adults in Ghana from an equity perspective, using a nationally representative sample. In addition, whilst most studies in the past have focused on horizontal equity, this study has clearly shown that due attention needs to be paid to both horizontal and vertical equity concepts to draw proper conclusions about where policy interventions need more focused attention.

A key limitation of this study is that causal inferences cannot be drawn from the findings due to its cross-sectional nature. Caution is thus advised regarding any broad policy conclusions or generalisations beyond the present study context. It is hoped that subsequent results from the SAGE longitudinal study would help to further investigate and concretely establish the nature of the relationships observed in this study.

Secondly, as is common with self-reported data, problems with recall and differential reporting by respondents could have introduced bias. Outpatient use was therefore limited to the preceding 12 months to minimise recall bias. A self-perceived need may also not reflect a true medical need at the time of the study [21] because self-rated health changes over time. Two determinants of healthcare need were thus used in the analysis to minimise the effect of this problem but the extent of their relevance in capturing true need could not be established. It would be interesting to observe what the findings would be with more objective measures for medical needs such as proven clinical diagnoses, in this population.

Because a majority of the study population were low educated, it is possible that there may have been knowledge limitations regarding their specific medical conditions, thereby resulting in either under or over-reporting. To partly address this, symptom-specific items in the questionnaire were included to identify some chronic medical conditions.

Additionally, factors that affect the use of healthcare services are diverse and complex. We could not have dealt with all the issues at hand in this study. For instance, factors such as socio-cultural practices [6], marital status and social support systems [18], the structure and organization of service delivery such as distance to care facilities and specific health financing arrangements [49] have not been the focus of this study. Additionally, this study focuses only on + 50 years age group without any comparison with the younger adults or children population when probably the major equity vault-lines in healthcare access lie between these major age groups.

## Conclusions

The current study is among the very few to have looked at how the concept of equity is operationalised in the use of healthcare among older persons in Ghana. It has shown that whilst inpatient healthcare access is equitable, disparities in SES are contributing greatly to inequity in accessing outpatient care and that older adults with the greatest health needs do not appropriately have more access to outpatient care.

The findings add to the growing debate about the need to ensure equitable access to healthcare services for all in the society especially for vulnerable groups like older adults. Measures that aim to reduce unfair disparities in healthcare access could go a long way in ensuring that older Ghanaians receive a fair opportunity to have their health needs addressed. This paper provides a strong starting point for further research into issues of equity in accessing healthcare among the older Ghanaian population. Further studies would be required to ascertain the impact that social protection initiatives have had on bridging the inequity gap among older persons and how health transitions affect the use of healthcare services. Regarding the latter, it is hoped that the WHO SAGE panel data availability will make this possible.

## Abbreviations

CI: Confidence interval
LMIC: Low- and middle-income countries
NHIS: National health insurance scheme
OR: Odds ratio
SAGE: Study on global AGEing and adult health
SES: Socioeconomic status
WHO: World Health Organization

## References

[1] World Health Organization. Priorities for research to take forward the health equity policy Agenda Report from the WHO Task Force on Health System Research Priorities for Equity in Health. Geneva: World Health Organization; 2004. PMC2626494 16462988

[2] World Health Organization. Global strategy for health for all by the year 2000. Geneva: WHO; 1981. Health for all series, 1985(3).

[3] Ministry of Health. National Health Insurance Policy Framework for Ghana. 2004; Available from: https://www.ghanahealthservice.org/downloads/NHI_policy%20framework.pdf. Accessed 6 Jan 2018.

[4] Department of Health. National-Health-Insurance-for-South-Africa-White-Paper.pdf. 2015; Available from: https://www.health-e.org.za/wp-content/uploads/2015/12/National-Health-Insurance-for-South-Africa-White-Paper.pdf. Accessed 15 May 2018.

[5] McIntyre D, Thiede M, Birch S (2009). Access as a policy-relevant concept in low- and middle-income countries. Health Econ Policy Law.

[6] Andersen RM (1995). Revisiting the behavioral model and access to medical care: does it matter?. J Health Soc Behav.

[7] Aday LA, Andersen RM (1981). Equity of access to medical care: a conceptual and empirical overview. Med Care.

[8] Wagstaff A, van Doorslaer E, Paci P. Horizontal equity in the delivery of health care. 1991. p. 251–6. 10.1016/0167-6296(91)90003-6 10113709

[9] Sutton M (2002). Vertical and horizontal aspects of socio-economic inequity in general practitioner contacts in Scotland. Health Econ.

[10] United Nations Department of Economic and Social Affairs. World population ageing. 2013; ST/ESA/SER.A/348. Available from: http://www.un.org/en/development/desa/population/publications/pdf/ageing/WorldPopulationAgeing2013.pdf. Accessed 7 May 2018.

[11] He, W. and P. Kowal, An Aging World: 2015, in International Population Reports. 2016: Washington D.C.

[12] Parmar D (2014). Enrolment of older people in social health protection programs in West Africa – does social exclusion play a part?. Soc Sci Med.

[13] Lambo E, Sambo LG (2003). Health sector reform in sub-Saharan Africa: a synthesis of country experiences. East Afr Med J.

[14] Schieber G, et al. Health financing in Ghana. Health financing in Ghana. 2012.

[15] Albanese E (2011). Equity in the delivery of community healthcare to older people: findings from 10/66 dementia research group crosssectional surveys in Latin America, China, India and Nigeria. BMC Health Serv Res.

[16] Ameh S (2014). Predictors of health care use by adults 50 years and over in a rural south African setting. Glob Health Action.

[17] Wandera SO, Kwagala B, Ntozi J (2015). Determinants of access to healthcare by older persons in Uganda: a cross-sectional study. Int J Equity Health.

[18] Roy K, Chaudhuri A (2008). Influence of socioeconomic status, wealth and financial empowerment on gender differences in health and healthcare utilization in later life: evidence from India. Soc Sci Med.

[19] Wong R, Díaz JJ (2007). Health care utilization among older Mexicans: health and socioeconomic inequalities. Salud Publica de Mexico.

[20] Guerra HL (2001). The Bambuí health and aging study (BHAS): factors associated with hospitalization of the elderly. Cadernos de Saude Publica.

[21] Peltzer K, et al. Universal health coverage in emerging economies: findings on health care utilization by older adults in China, Ghana, India, Mexico, the Russian Federation, and South Africa. Glob Health Action. 2014;7. 10.3402/gha.v7.25314. 10.3402/gha.v7.25314 PMC4216816 25363363

[22] Government of Ghana. National ageing policy. ‘Ageing with security and dignity’. 2010; Available from: http://mogcsp.gov.gh/mdocs-posts/national-ageing-policy-ageing-with-security-and-dignity/. Accessed 6 May 2018.

[23] Wang H, Otoo N, Dsane-Selby L (2017). Ghana National Health Insurance Scheme: improving financial sustainability based on expenditure review.

[24] Nyonator F, Kutzin J (1999). Health for some? The effects of user fees in the Volta region of Ghana. Health Policy Plan.

[25] National Health insurance Authority. National health insurance scheme, annual report 2013. 2013; Available from: http://www.nhis.gov.gh/files/2013%20Annual%20Report-Final%20ver%2029.09.14.pdf. Accessed 6 Apr 2018.

[26] Adisah-Atta I (2017). Financing health Care in Ghana: are Ghanaians willing to pay higher taxes for better health care? Findings from Afrobarometer. Soc Sci.

[27] Alhassan RK, Nketiah-Amponsah E, Arhinful DK (2016). A review of the National Health Insurance Scheme in Ghana: what are the sustainability threats and prospects?. PLoS One.

[28] Mills A (2012). Equity in financing and use of health care in Ghana, South Africa, and Tanzania: implications for paths to universal coverage. Lancet.

[29] Odeyemi IA, Nixon J (2013). Assessing equity in health care through the national health insurance schemes of Nigeria and Ghana: a review-based comparative analysis. Int J Equity Health.

[30] Garshong, B. and B. Akazili, Universal health coverage assessment: Ghana. 2015, Global Network for Health Equity (GNHE).

[31] Akazili J (2014). Is Ghana’s pro-poor health insurance scheme really for the poor? Evidence from northern Ghana. BMC Health Serv Res.

[32] Government of Ghana. Livelihood empowerment against poverty. 2016; Available from: http://leap.gov.gh/. Accessed 6 Apr 2018.

[33] Saeed BI (2016). Effect of socio-economic factors in utilization of different healthcare services among older adult men and women in Ghana. BMC Health Serv Res.

[34] Saeed BI (2015). Impact of socioeconomic status and medical conditions on health and healthcare utilization among aging Ghanaians. BMC Public Health.

[35] Vallejo-Torres L, Morris S (2013). Income-related inequity in healthcare utilisation among individuals with cardiovascular disease in England-accounting for vertical inequity. Health Econ.

[36] Kowal P (2012). Data resource profile: the World Health Organization study on global AGEing and adult health (SAGE). Int J Epidemiol.

[37] Biritwum RB, et al. Household characteristics for older adults and study background from SAGE Ghana wave 1. Glob Health Action. 2013;6. 10.3402/gha.v6i0.20096. 10.3402/gha.v6i0.20096 PMC3681208 23759325

[38] Grundy E, Holt G (2001). The socioeconomic status of older adults: how should we measure it in studies of health inequalities?. J Epidemiol Community Health.

[39] DeSalvo KB (2005). Predicting mortality and healthcare utilization with a single question. Health Serv Res.

[40] Sanderson D, et al. Perceived health. Selection of a coherent set of health indicators for European Union. Montpellier: Euro-REVES, 2002: p. 129.

[41] Martikainen P (1999). Reliability of perceived health by sex and age. Soc Sci Med.

[42] Archer KJ, Lemeshow S, Hosmer DW (2007). Goodness-of-fit tests for logistic regression models when data are collected using a complex sampling design. Computational Statistics and Data Analysis.

[43] Phelan JC, Link BG, Tehranifar P (2010). Social conditions as fundamentalcCauses of health inequalities: theory, evidence, and policy implications. J Health Soc Behav.

[44] Terraneo M (2015). Inequities in health care utilization by people aged 50+: evidence from 12 European countries. Soc Sci Med.

[45] World Health Organization. Ghana country assessment report on ageing and health. 2014; Available from: http://www.who.int/ageing/publications/ghana/en/. Accessed 6 May 2018.

[46] Shi L (2002). Primary care, self-rated health, and reductions in social disparities in health. Health Serv Res.

[47] Minicuci N, et al. Sociodemographic and socioeconomic patterns of chronic non-communicable disease among the older adult population in Ghana. Glob Health Action. 2014;7. 10.3402/gha.v7.21292. 10.3402/gha.v7.21292 PMC3991840 24746141

[48] Gatimu SM, Milimo BW, Sebastian MS (2016). Prevalence and determinants of diabetes among older adults in Ghana. BMC Public Health.

[49] Andersen R, Davidson P, Baumeister S. In: Kominski GF, editor. Improving access to care, in Changing the U. S. health care system. Somerset: Wiley; 2013. p. 33–69.

